# Patients With Voice Prosthesis Rehabilitation During the COVID-19 Pandemic: Analyzing the Effectiveness of Remote Triage and Management

**DOI:** 10.1177/0194599820948043

**Published:** 2020-08-04

**Authors:** Ylenia Longobardi, Jacopo Galli, Lucia D’Alatri, Vezio Savoia, Giorgia Mari, Mario Rigante, Giulio Cesare Passali, Francesco Bussu, Claudio Parrilla

**Affiliations:** 1 Unità Operativa Complessa di Otorinolaringoiatria, Area Testa-Collo, Dipartimento Scienze dell’Invecchiamento, Neurologiche, Ortopediche e della Testa-collo, Fondazione Policlinico Universitario A. Gemelli, IRCCS, Rome, Italy; 2 Servizio di Psicologia, Fondazione Policlinico Universitario A. Gemelli IRCCS, Rome, Italy; 3 ENT Division, Azienda Ospedaliero Universitaria, Sassari, Italia

**Keywords:** total laryngectomy, COVID-19, voice prosthesis, management

## Abstract

**Objective:**

To describe a remote approach used with patients with voice prosthesis after laryngectomy during the COVID-19 pandemic and the resulting clinical outcomes in terms of voice prosthesis complications management, oncological monitoring, and psychophysical well-being.

**Study Design:**

Prospective cohort study.

**Setting:**

Otolaryngology Clinic of the University Polyclinic A. Gemelli, IRCCS Foundation.

**Subjects and Methods:**

All patients with voice prosthesis who underwent laryngectomy followed by our institute were offered enrollment. Patients who agreed to participate were interviewed to inquire about the nature of the need and to plan a video call with the appropriate clinician. Before and 1 week after the clinician’s call, patients were tested with the Hospital Anxiety and Depression Scale. Degrees of satisfaction were investigated with a visual analog scale. A comparison between those who accepted and refused telematic support was carried out to identify factors that influence patient interest in teleservice.

**Results:**

Video call service allowed us to reach 37 (50.68%) of 73 patients. In 23 (62.16%) of 37 cases, the video call was sufficient to manage the problem. In the remaining 14 cases (37.83%), an outpatient visit was necessary. Participants who refused telematic support had a significantly shorter time interval from the last ear, nose, and throat visit than patients who accepted (57.95 vs 96.14 days, *P* = .03). Video-called patients showed significantly decreased levels of anxiety and depression (mean Hospital Anxiety and Depression Scale total score pre– vs post–video call: 13.97 vs. 10.23, *P* < .0001) and reported high levels of satisfaction about the service.

**Conclusion:**

Remote approach may be a viable support in the management of patients with voice prosthesis rehabilitation.

On March 11, 2020, the World Health Organization stated that a new coronavirus for severe acute respiratory syndrome, called SARS-CoV-2 and identified as a microbial agent that causes viral pneumonia, could be characterized as a pandemic.^1^ Initially linked to Wuhan (Hubei province, China), coronavirus disease (COVID-19) has progressively involved many countries, including Italy with 204,576 confirmed cases and 26,049 deaths according to the data of the Istituto Superiore di Sanità on May 1, 2020.^2^

All over the world, the most affected countries have faced new multifaceted issues. To limit viral spread, the Italian government has put into place extraordinary measures that culminated on March 9 with a lockdown that inhibited the movement of people. The most fragile patients and those with anatomic or surgical alterations of the upper airway seem to be the most prone to contagion. In addition, the risk of infection is higher in the hospital setting than in the community: it has been estimated that hospital-related transmission occurs in >40% of cases.^3^ For this reason, patients requiring periodic visits represent a complicated task for clinicians during this critical period. Against this background, patients who underwent laryngectomy are a unique challenge for clinical management because of the interruption between the upper airway and the trachea and the complete respiratory dependence through the tracheostoma. The nonpassage of air in the nasal cavities and the consequent loss of the filtering function place such patients in a condition of greater risk of inhaling pathogens and developing respiratory infections. This implies the need for greater caution during the current epidemic.^4-7^

In addition, the risk of poor outcomes with COVID-19 is higher in patients who underwent a laryngectomy because of the frequent presence of medical comorbidities (ie, chronic lung disease, peripheral vascular disease, heart disease, cerebrovascular disease, and diabetes), history of smoking, and impairment of mucociliary function by inhalation of cold and dry air.^8,9^

Finally, most of these patients undergo rehabilitation with a voice prosthesis (VP), which allows the acquisition of a fluent, sonorous voice with good prosody and intelligibility; therefore, it is considered the gold standard.^10-12^ Nevertheless, it obliges the patient to undergo periodic outpatient visits for natural wear and tear over time or malfunctions, which represent a safety risk,^13^ or receive an annual medical prescription of aids for pulmonary and phonatory rehabilitation.

For all the aforementioned reasons, the patient who undergoes a laryngectomy is typically considered a “fragile” or “demanding” patient. Anatomic-functional changes negatively affect the patient’s quality of life, and the registered psychological trauma is often more intense and significant than that found in patients with tumors of other sites.^4,14^ Psychopathologic symptoms, such as depression and anxiety, are present in at least 30% of such patients,^15-17^ and it is common for them to establish a relationship of close dependence with their ear, nose, and throat (ENT) doctor and speech therapist.

Currently, the Otolaryngology Clinic of the University Polyclinic A. Gemelli, IRCCS Foundation, manages 84 patients who underwent a laryngectomy with VP in a multidisciplinary path that includes an ENT surgeon, speech therapist, phoniatrician, and psycho-oncologist. The heterogeneity of clinical manifestations and complications affecting these patients requires periodic multidisciplinary evaluations to investigate the issue and verify the appropriateness of a diagnostic-therapeutic program. On average, we record 250 visits per year by these patients.

The potential threat of COVID-19 for our patients has reversed the usual risk-benefit balance. Clinicians have to consider, on a case-by-case basis, the possibility of postponing nonurgent outpatient activities,^18^ and patients increasingly prefer to avoid coming to the hospital, thereby risking a higher rate of VP-related complications or delay in tumor recurrence diagnosis.

For all these reasons, we have proposed a remote approach (via email, by phone, or via Skype) to evaluate patients’ overall conditions and to intervene before the occurrence of emergencies, the resolutions of which would require a visit and/or hospitalization.

The aim of this article is to describe the approach used and the clinical results in terms of VP complications management, oncologic monitoring, and psychophysical well-being of the patients evaluated before and after the telematic support.

## Materials and Methods

### Patients and Study Design

This is a prospective cohort study including all patients who underwent a laryngectomy with VP and were followed in the Otolaryngology Clinic of our institute. Our ethical committee approved the study (Comitato Etico Fondazione Policlinico Universitario “Agostino Gemelli” IRCCS, Università Cattolica del Sacro Cuore; 3181).

Adult patients (N = 84) fitting inclusion criteria (laryngectomy with VP, able to provide written informed consent) were selected and contacted via email or telephone to propose enrollment and explain the service. Patients who agreed to participate were recontacted, and an online informed consent form was sent them. Patients were tested with the Hospital Anxiety and Depression Scale (HADS), and a semistructured interview was conducted to inquire about the nature of the need (VP-related trouble, medical/surgical issue, or psychological aid). On the basis of the answers obtained, patients were contacted, via telephone or Skype, by the ENT doctor, speech therapist, and/or psycho-oncologist to evaluate the presence and severity of oncologic or VP complications, as well as the state of psychophysical health. If needed, a subsequent outpatient visit was planned. Our COVID-19 point-of-care protocol before VP replacement included a self-declaration form (see Supplemental Appendix A, available online), gloves, and FFP2 mask for patients; a temperature check at the service entrance with an infrared thermometer; and a direct path to a dedicated outpatient room without staying in the waiting room.

The COVID-19 protocol for the staff during VP management provided for the involvement of only 2 clinicians (ENT surgeon and speech therapist) wearing an FFP2 mask, protective glasses, disposable water-repellent gown, disposable shoe covers, helmet with transparent visor, and gloves (2 pairs with alcoholic gel in the middle).

To avoid any contamination after the clinician’s decision about the size of the new VP, an external nurse procured it. At the end of the procedure, sanitization of the armchair and room was performed, and an interval of 2 hours before the next visit was always respected.

One week after the clinician’s call, patients were contacted again via email to readminister the HADS and to investigate degrees of satisfaction. Patient satisfaction about service provided was evaluated by a visual analog scale (VAS).

**Figure 1.**
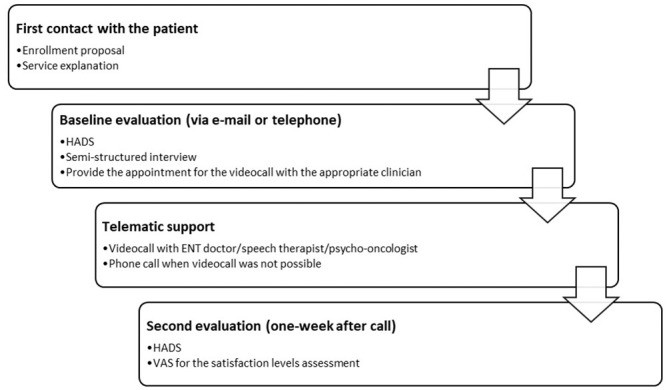
Flow diagram of study steps. ENT, ear, nose, and throat; HADS, Hospital Anxiety and Depression Scale; VAS, visual analog scale.

The flow diagram of study steps is shown in **Figure 1**.

### Telematic Support

The video calls were performed by the referring ENT doctor, speech therapist, or psycho-oncologist and took place from the workstations of the Otolaryngology Clinic of the University Polyclinic A. Gemelli, IRCCS Foundation. The Skype platform or video call function of WhatsApp was used to connect with patients remotely. For patients who were not technologically savvy or had issues accessing telehealth, the aid of family members was requested.

During the video calls, information was provided on how to better protect the lower airways, the hygienic precautions to take during the management of the VP and the tracheostoma, and the personal protective equipment to be worn. Any problems related to the VP were discussed to reserve outpatient visits—and, therefore, patient access to the hospital—only for urgencies that could not be postponed.

To prepare the patients, we asked them to have ready-made cleaning devices on hand, such as the brush and flush, an aspirator, and a glass of water to check for leakages. Patients who delayed or canceled their oncologic follow-up showed the results of blood tests, computed tomography scans, and magnetic resonance imaging to doctors. The annual prescriptions of aids were discussed with patients during the video calls and then sent via email.

**Figure 2.**
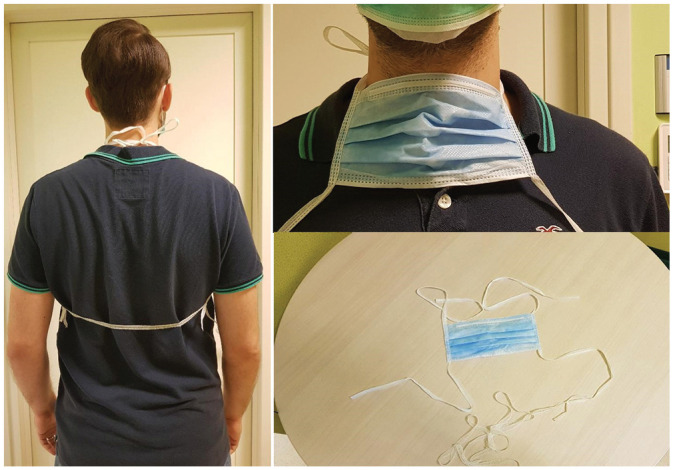
Example on the correct way to wear the mask for the tracheostomy protection.

**Figure 3.**
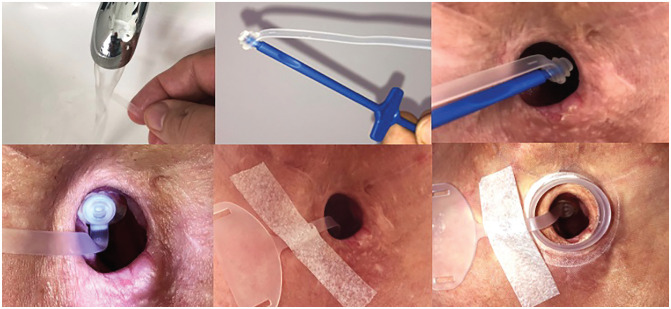
Step-by-step scheme for using the plug. Courtesy of Atos Medical.

Patients were also allowed to discuss with a specialist with any fears and emotional discomforts associated with the particular period that they were experiencing. After the video call, visual support such as drawings or tools was often sent to the patients to help them better understand the advice (**Figures 2** and **3**).

### Outcome

#### Hospital Anxiety and Depression Scale

The HADS is a self-report questionnaire used to assess anxiety and depressive symptoms in a general medical population.^19,20^ It consists of 14 items: 7 items to evaluate anxiety and 7 to evaluate depression. Each item is rated on a 4-point scale that goes from a minimum of 0 (never) to a maximum of 3 (always), depending on how often the experience occurs. The total score can vary from 0 to 21 in each subscale. According to the literature, the cutting point adopted is a score ≥8 to indicate anxiety (HADS-A) and a score ≥9 to indicate depression (HADS-D).

#### VAS for Satisfaction

The VAS is a horizontal line 10 cm long. At the beginning and at the end, there are 2 descriptors representing extremes of satisfaction (ie, no satisfaction and extreme satisfaction). The patient rates satisfaction by making a vertical mark on the 10-cm line. The measurement in centimeters is converted to the same number of points ranging from 0 to 10. The exact question is “Are you satisfied with the received video call?” This instrument has been considered sensible, simple, reproducible, and universal. A standard explanation of how to fill in the VAS form is mentioned beneath the horizontal line.^21^

### Statistical Analysis

We used the statistical package MedCalc (v 12). The Kolmogorov-Smirnov test was used to assess the distribution of the continuous variables examined in the study. Parametric tests were applied depending on the data distribution. The chi-square test was used to compare categorical data. Significance was accepted for *P* values <.05.

## Results

We initially selected 84 patients. It was not possible to contact 9 of them because of absent email contact or wrong telephone number. Two patients were excluded because of unexpected death (1 for heart attack and 1 for acute cerebral hemorrhage) after the beginning of the study. The definitive sample therefore included 73 patients who underwent a laryngectomy (68 men and 5 women; mean ± SD age, 68.34 ± 9.50 years; range, 31-83 years). All patients had undergone total laryngectomy with bilateral neck dissection and tracheoesophageal puncture (TEP) for voice restoration. All patients had a primary closure of the pharynx, except for 10 free flap nontubed reconstructions (8 anterolateral thigh and 2 forearm). Of 73 patients, 48 have been subjected to radiotherapy: primary (10/48, 13.69%) or adjuvant (38/48, 52.05%). In 27 (36.98%) of 73 patients, a secondary TEP was performed. The interval between total laryngectomy and prosthesis implantation varied from 6 to 48 months (mean, 22.90 ± 25.80).

Among the 73 contacted patients, 36 (49.31%) did not refer any trouble, and they decided to not perform the video call. A total of 37 (50.68%) patients were called in the period between April 7 and May 11, 2020. Characteristics of 2 groups of patients were summarized in **Table 1**.

**Table 1.** Characteristics of Patients Who Accepted and Refused the Telematic Support.

	Patients, No.	
	Accepted (n = 37)	Refused (n = 36)
Male:female	35:2	33:3
Age,^a^ y	67.72 ± 9.20	67.94 ± 9.79
Radiotherapy		
Primary	9	1
Adjuvant	19	19
None	9	16
Puncture		
Primary	24	22
Secondary	13	14

a Mean ± SD.

### Telematic Support

Of 37 patients, 14 (37.83%) requested the video call to receive a medical/speech therapy consultation, 18 (48.64%) for VP-related issues (**Table 2**), and 5 (13.51%) to have a psychological consultation. Examples of advice given to patients are listed in **Table 3**.

**Table 2.** Voice Prosthesis Troubles Experienced by Patients Who Required Video Call.

	Voice prosthesis issue	
	Video called (n = 18)	Required in-person visit (n = 12)
Leakage		
Intravalvular	8	5
Periprosthetic	6	5
Granuloma	3	1
Extrusion	1	1

**Table 3.** Examples of Advice Given to Patients.

Minimize aerosolized particle spread	Cover the tracheostoma with an HME, preferably equipped with an electrostatic filter (Provox Micron HME).
	Put a physical barrier over the stoma, such as a surgical mask.
Reduce the risk of contagion	Wear surgical mask over mouth, nose, and stoma with HME.
	Tie upper mask strings around neck; use additional extension string to connect the 2 lower mask strings under the arms and behind the back.
	Wear hands-free HME because it does not require touching when speaking, or wash hands as much as possible before touching regular HME or managing the stoma. Wash hands with soap and water for at least 20 s or an alcohol-based hand sanitizer with at least 60% alcohol.
Voice prosthesis leaking	Clean and rotate the voice prosthesis.
	Train to use the plug to contain intravalvular leakage.
	Suggest alternative dietary measures, such as thickened liquids.

Abbreviation: HME, heat and moisture exchanger.

In 23 cases (62.16%), the video call was sufficient to manage the problem; in the remaining 14 cases (37.83%), an outpatient visit was necessary (12 VP replacement, 2 diagnostic tests). Twelve annual prescriptions were sent during the selected period.

**Figure 4.**
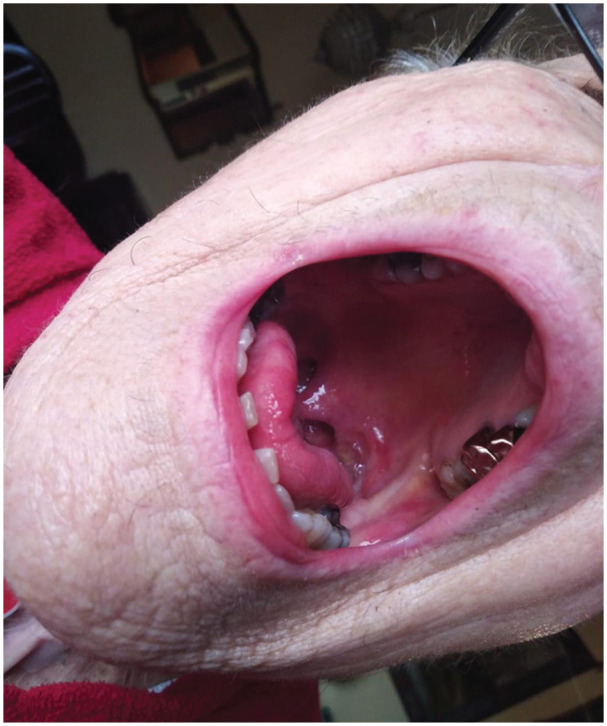
Soft palate mucosal ulceration found during a video call, later diagnosed as tonsillar pillar tumor.

During the video medical consultation, 2 cases of tumor recurrence/second primary tumor were diagnosed. Interestingly, in 1 patient who complained of slight pain in the oral cavity, the surgeon detected a small mucosal ulceration by targeting the camera on the soft palate (**Figure 4**). In the second patient, complaining of VP dislocation, the camera showed a slight bulging on the right tracheal lateral wall with a preserved mucosal layer. In both cases, a subsequent outpatient visit was planned, and after biopsy and computed tomography scan, a second primary tumor and a peristomal recurrence were detected, respectively.

During the psychological consultation, it was possible to early detect 3 cases of severe anxiety-depressive disorder, and online psychotherapy was started. Two patients who underwent surgery and were discharged during the lockdown and never performed speech therapy used the VP for the first time during the video call. The first postoperative visit, usually 2 weeks after discharge, was avoided and performed via video call. For these patients, telerehabilitation was delivered.

**Figure 5.**
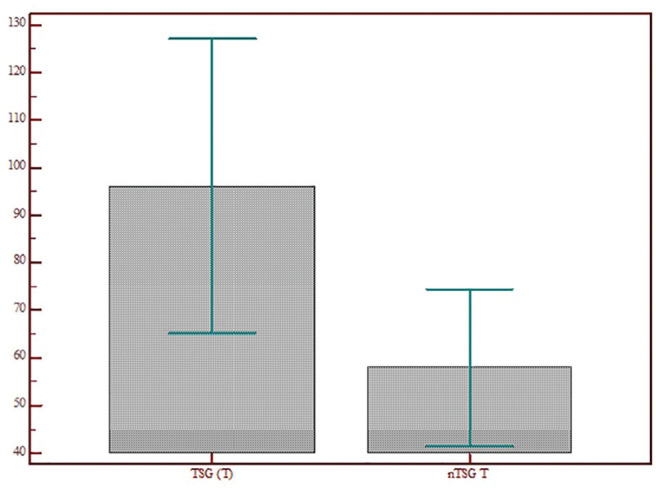
Comparison between patients who accepted telematic support and those who refused in terms of difference of time interval (days) from last ear, nose, and throat visit. nTSG T, no telematic support group time; TSG T, telematic support group time. Values are presented as mean ± SD

Comparison between patients who accepted telematic support and those who refused showed a statistically significant difference of time interval (days) from last ENT visit: patients who took part in the study had a longer interval than those who did not (96.14 vs 57.95 days, *P* = .03; **Figure 5**). Instead, no significant difference in age, time elapsed from the puncture, or type of puncture (primary vs secondary) was found between the groups (*P* > .05).

### Hospital Anxiety and Depression Scale

**Figure 6.**
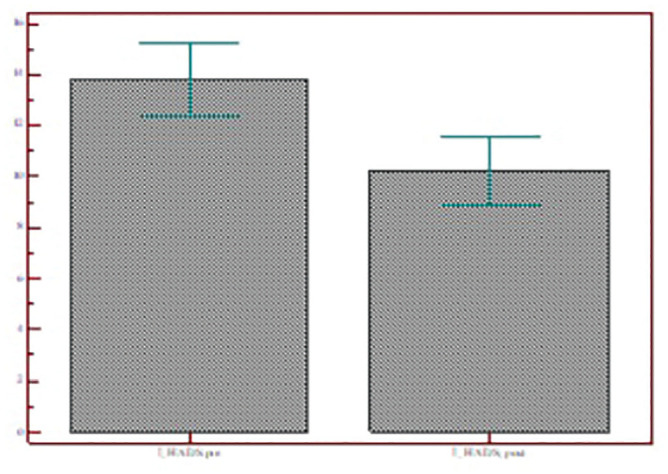
Total HADS scores of patients who accepted telematic support before and after the video call. Values are presented as mean ± SD. HADS, Hospital Anxiety and Depression Scale.

**Figure 7.**
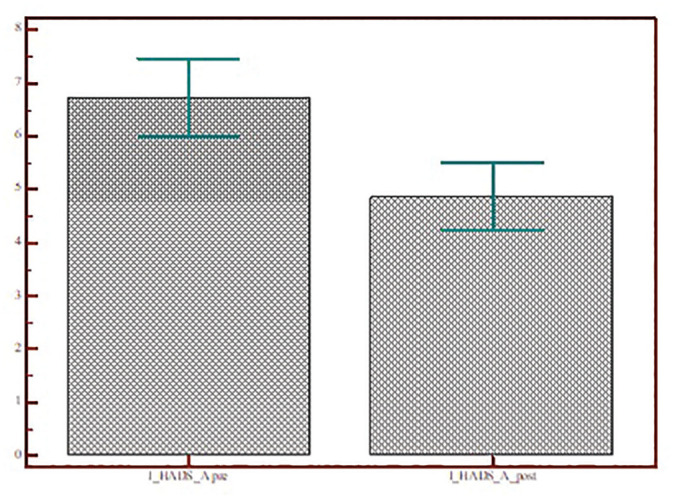
Scores for the HADS Anxiety subscale of patients who accepted telematic support before and after the video call. Values are presented as mean ± SD. HADS, Hospital Anxiety and Depression Scale.

**Figure 8.**
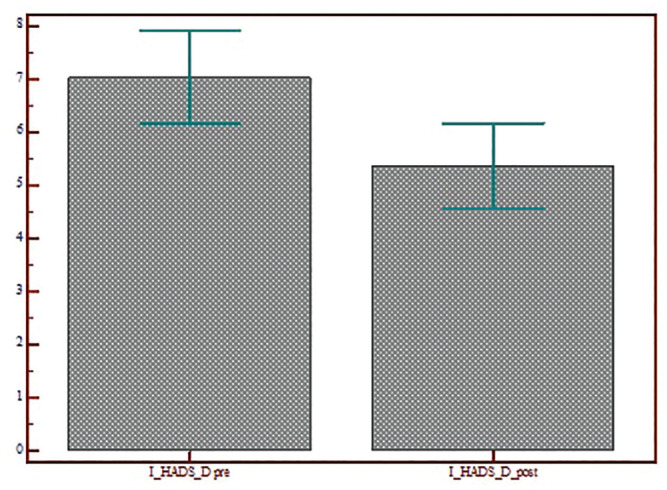
Scores for the HADS Depression subscale of patients who accepted telematic support before and after the video call. Values are presented as mean ± SD. HADS, Hospital Anxiety and Depression Scale.

The Kolmogorov-Smirnov test showed that the outcome variable HADS had a normal distribution. A 2-sided Student’s *t* test was used to analyze the HADS score pre– and post–telematic support. The scores decreased significantly in all subtests: HADS (13.97 ± 9.01 vs 10.23 ± 8.16, *P* < .0001), HADS-A (6.94 ± 4.65 vs 4.86 ± 3.91, *P* < .0001), and HADS-D (6.97 ± 5.31 vs 5.36 ± 4.93, *P* = .0001; **Figures 6**-**8**).

### VAS for Satisfaction

Of 37 patients, 31 (83.78%) reported a score of 10 at the VAS. The remaining 6 patients reported a score of 9 (n = 1, 2.70%) and 8 (n = 5, 21.62%).

## Discussion

The COVID-19 pandemic has presented learning opportunities for cancer centers. A new analysis estimates that a 20% increase of deaths should occur over the next 12 months in patients with cancer diagnoses because of people with cancer who contract COVID-19 or receive delayed oncologic diagnosis or treatment.^22^

In this setting, patients who underwent a laryngectomy represent a specific difficult task because of the higher potential risk of mortality from COVID-19 due to concomitant respiratory comorbidities and the transmission of viral particles due to direct aerosolization from the tracheostoma. In particular, patients who have undergone rehabilitation with a VP represent a unique subset given their demanding management. In fact, the laryngectomy guidance of the British Association of Head and Neck Oncologists during the COVID-19 pandemic recommends avoiding a primary TEP, preferring a secondary one performed at a later date.^23^

In our opinion, decreasing the level or postponing the onset of rehabilitation is not the solution, especially because the trajectory of this pandemic is uncertain and we must prepare to live with it. In this scenario, we must recognize that the management of “new” and “old” patients will have to be modified and adapted to the current COVID-19 crisis. Our goal remains to provide high-quality care under circumstances that we have never had to face, as safe as possible for patients with cancer and for staff.

Concern about the exposure of patients to COVID-19 has grown with the spread of the virus, and difficult decisions on how and when to provide treatment have become a necessity. In agreement with several authors,^8,24-26^ we decided that every checkup for existing patients could be switched to telemedicine. For this reason, we quickly expanded our telemedicine efforts. The multidisciplinary team has increased its acute assessment skills to ensure that hospital access is reserved only to those who need higher-level care. Clinician-to-patient calls have optimized shared decision making to delay outpatient visits. Video call service has been requested by 37 of 73 (50.68%) patients. The analysis of the differences between the group that requested the remote support and the group that did not showed that the time since the last visit was the only parameter that influenced the decision. Patients who have not visited for >2 months have joined the service, thereby demonstrating that remote management could be a valid support in ensuring the continuity of care that patients need.

Of 37 patients, 32 (86.48%) requested the video call for clinical reasons (medical/speech therapy consultation, VP-related issues) and 5 (13.51%) for psychological support. We consider both reasons of equal importance, as the common goal of the clinicians is to avoid unnecessary hospital accesses. Moreover, patients who underwent a laryngectomy and are isolated during this period, per the advice of national and local government, may not be able to understand when access to the hospital is necessary. Not knowing when the risk of coming to the hospital is justified or not can generate a crisis and increase the anxiety and depression levels typical of these patients. Providing virtual checks just to see how patients are doing helps them feel monitored. This concept is reinforced by the statistically significant decrease found in the scores obtained by patients at HADS before and after the video call.

Of 37 patients remotely managed, 23 (62.16%) resolved their problem without coming to the hospital. Fourteen cases could not be postponed: 12 patients had to change their VP, with outpatient visits organized per our safety regulations, and 2 patients were hospitalized, one with a diagnosis of second primary tumor of the tonsillar pillar and the other with peristomal recurrence. In the latter cases, a real close-up and correct shot of the tracheostoma was key to understanding the problem. For this reason, we recommend the presence of a family member and the use of a phone (rather than a computer), which can better target the camera. Moreover, even the less “digital” patients are more likely to have a phone and a family member available to join in the telematic session.

In our study, we did not analyze the economic issue. Our protocol was built as a rapid response to fulfill the request of health care for “fragile patients,” and for this reason, no fee was provided for the service/hospital. In our country, the payment system for telemedicine is not uniform in all regions, but in light of the degree of satisfaction found in patients, we believe that local administrations should provide for a reimbursement system that allows the use of virtual visits even after the COVID-19 emergency. Future studies to establish the cost-effective ratio of the telemedicine should better establish which patients can benefit from a remote management, keeping intact the safety and sensitivity of our consult for medicolegal reasons as well.

In conclusion, the results of our prospective study showed that televisits reduce anxiety/depression in patients who underwent a laryngectomy when access to in-person clinical care was restricted. Furthermore, televisits facilitated effective management of medical needs during this time for a tertiary referral center that manages many patients, even from distant regions. Nowadays there is a lack of published efficacy studies for telemedicine as a component of TEP management. The protocol described provides evidence about the feasibility of telemedicine for this population, but further studies should validate the efficacy of such an approach to improve its potential use.

## Supplemental Material

APPENDIX_A – Supplemental material for Patients With Voice Prosthesis Rehabilitation During the COVID-19 Pandemic: Analyzing the Effectiveness of Remote Triage and Management Click here for additional data file. Supplemental material, APPENDIX_A for Patients With Voice Prosthesis Rehabilitation During the COVID-19 Pandemic: Analyzing the Effectiveness of Remote Triage and Management by Ylenia Longobardi, Jacopo Galli, Lucia D’Alatri, Vezio Savoia, Giorgia Mari, Mario Rigante, Giulio Cesare Passali, Francesco Bussu and Claudio Parrilla in Otolaryngology–Head and Neck Surgery
