# Supplementary material for: Patients With Voice Prosthesis Rehabilitation During the COVID-19 Pandemic: Analyzing the Effectiveness of Remote Triage and Management
Source: Otolaryngol Head Neck Surg. 2020 Aug 4;164(2):277–84. doi: 10.1177/0194599820948043 (PMC7404089; doi:10.1177/0194599820948043)
Supplement: APPENDIX_A – Supplemental material for Patients With Voice Prosthesis Rehabilitation During the COVID-19 Pandemic: Analyzing the Effectiveness of Remote Triage and Management [file APPENDIX_A.docx]

APPENDIX A

**ANAMNESTIC SELF-DECLARATION FORM**

I, the undersigned, _____________________________________ born in_____________________ _______________________on_____________________________, based in __________________ ________________________________________________________________________________ aware of the consequences of false declarations, I declare that:

1. I have no respiratory symptoms (cough, fever, sore throat, conjunctivitis, loss of smell and taste)
2. I had not respiratory symptoms (or diarrhea) in the 14 days prior to this access
3. I have filled in the declaration pursuant to the Ordinance of the President of the Regional Council of Lazio (n ° 4 / 8.03.2020)

I also declare that I am aware that this declaration will be included in the medical record.

Finally, I declare that I am aware that, regardless of my health conditions, I will have to wear the surgical mask for the entire duration of my stay in the hospital.

Date

____________________________

Signature

_______________________________________________________________________
